# Multifunctional Biotemplated Micromotors for In Situ Decontamination of Antibiotics and Heavy Metals in Soil and Groundwater

**DOI:** 10.3390/nano13192710

**Published:** 2023-10-06

**Authors:** Haohao Cui, Ke Wang, Enhui Ma, Hong Wang

**Affiliations:** School of Chemical Engineering and Technology, China University of Mining and Technology, Xuzhou 221116, China

**Keywords:** micromotors, biotemplated fabrication, water/soil treatment, antibiotics, heavy metal ions

## Abstract

The ubiquitous pollution by antibiotics and heavy metal ions has posed great threats to human health and the ecological environment. Therefore, we developed a self-propelled tubular micromotor based on natural fibers as an active heterogeneous catalyst for antibiotic degradation and adsorbent for heavy metal ions in soil/water. The prepared micromotors can move in the presence of hydrogen peroxide (H_2_O_2_) through a bubble recoil mechanism. The MnO_2_ NPs and MnFe_2_O_4_ NPs loaded on the hollow fibers not only enabled self-driven motion and magnetic control but also served as activators of peroxymononsulfate (PMS) and H_2_O_2_ to produce active free radicals SO_4_^•−^ and •OH. Benefiting from the self-propulsion and bubble generation, the micromotors can effectively overcome the disadvantage of low diffusivity of traditional heterogeneous catalysts, achieving the degradation of more than 90% TC in soil within 30 min. Meanwhile, due to the large specific surface area, abundant active sites, and strong negative zeta potential, the micromotors can effectively adsorb heavy metal ions in the water environment. In 120 min, self-propelled micromotors removed more than 94% of lead ions, an increase of 47% compared to static micromotors, illustrating the advantages of on-the-fly capture. The prepared micromotors with excellent catalytic performance and adsorption capacity can simultaneously degrade antibiotics and adsorb heavy metal ions. Moreover, the magnetic response enabled the micromotors to be effectively separated from the system after completion of the task, avoiding the problem of secondary pollution. Overall, the proposed micromotors provide a new approach to the utilization of natural materials in environmental applications.

## 1. Introduction

With the rapid progress of industrialization, environmental problems such as water and soil pollution also arise. Organic pollutants and heavy metal ions are the most common contaminants in soil and groundwater [1,2]. Among organic pollutants, antibiotics, widely used to treat various diseases of humans and animals, have become an emerging pollutant due to their overuse and stable nature, which constitute a threat to ecological safety. The wide existence of antibiotics in soil and water all over the world reveals the insufficiency of current methods for the removal of antibiotics [3,4]. Meanwhile, anthropogenic activities, e.g., metal-based industries and improper waste disposal, have led to the release of heavy metal ions into the environment. The toxic, bioaccumulative, and non-biodegradable characteristics of heavy metals have aroused extensive concern in terms of the environment and public health [5,6,7,8]. Although a variety of treatment methods have been proposed to remove these pollutants, efficient remediation techniques are still urgently needed to cope with the pollution situation.

As a less disruptive approach that is cost-effective and relatively rapid, in situ remediation is considered to be a promising technology in the treatment of contaminated soil and groundwater and tremendous progress has been made toward the removal of a spectrum of pollutants [9]. In situ chemical oxidation (ISCO) technology is commonly used to degrade organic pollutants such as polycyclic aromatic hydrocarbons and antibiotics existing in soil [10]. This method can degrade the pollutants on-site by injecting strong oxidants into contaminated soil. However, the degradation effect of ISCO is limited by the low diffusivity of catalysts in the heterogeneous soil environment, which hinders the transport of catalysts to areas distant from the injection point. In addition, adsorption is widely employed in the removal of heavy metal ions in water because of its simple operation, flexible nature, and strong stability [11,12]; however, the low diffusivity of adsorbents also affects the in situ removal efficiency of heavy metals in practical applications [13]. As such, the limited transport of catalyst and adsorbents constitutes a main problem in in situ remediation technology and therefore, it is urgent to develop new materials to solve the dilemma.

Self-propelled micromotors have attracted extensive interest from researchers around the world in the field of environmental remediation [14,15]. The excellent performance of micromotors also opens new horizons in the field of environmental remediation [16]. Micromotors able to convert external energy into self-driven motion can enhance the mixing of surrounding fluids and generate intensified mass transfer through their own movement and bubbles, which hold great potential in solving the disadvantages of the low diffusivity of traditional heterogeneous catalysts and adsorbents [17,18,19]. Whereas the use of expensive materials and the complex preparation process of micromotors make it difficult to apply them in large-scale practical applications [20,21,22], the utilization of plant materials with natural regular structures as templates provides a new way for the large-scale and low-cost manufacturing of micromotors. Furthermore, the replacement of noble metal catalysts in micromotors with other cost-effective catalysts also contributes to a reduction in fabrication costs.

In this work, we have developed tubular micromotors based on Kapok fibers as active heterogeneous catalysts for the degradation of antibiotics and adsorbents for heavy metal ions. The micromotors were prepared by the modification of MnO_2_ NPs and MnFe_2_O_4_ NPs on the hollow fibers. The MnO_2_ NPs can catalyze H_2_O_2_ to produce oxygen bubbles to achieve the motion of the micromotors via bubble recoil. The enhancement of mass transfer generated by the micromotors in the process of autonomous motion can facilitate the transport of heterogeneous catalysts in soil and water in in situ remediation. In addition, the MnFe_2_O_4_ NPs together with MnO_2_ NPs serve as catalysts of Fenton-like reactions to produce SO_4_^•−^ and •OH. The large specific surface area of the natural tubular fibers, a large number of active sites, and the strong negative zeta potential on the surface of the micromotors allow their efficient adsorption of lead. It is also worth mentioning that MnFe_2_O_4_ NPs also impart magnetic responsiveness to the micromotors, which facilitates their subsequent recovery. The proposed micromotors based on kapok fibers (KFs) as active heterogeneous catalysts of advanced oxidation processes and adsorbents of heavy metals in in situ remediation provide a new platform for the treatment of pollutants in soil and groundwater.

## 2. Experimental

### 2.1. Materials

Hydrogen peroxide (H_2_O_2_, AR, 30 wt%) and potassium permanganate (KMnO_4_, AR, 99%) were purchased from Xilong Scientific (CXL) (Shantou, China). Ethylene glycol (AR, 99%), polyethylene glycol (AR, 99%), McIlvaine-Na_2_EDTA (≥99.0%), potassium persulfate (PMS, 99%), and TBA (AR, ≥99%) were purchased from Innochem (Gwinnett Village, GA, USA). Sodium hydroxide (NaOH, AR, 96%), SDS (ACS, ≥99.0%), acetonitrile (AR, ≥99.9%), sodium acetate anhydrous (NaAc, AR, 99.99%), and manganese chloride tetrahydrate (MnCl_2_·4H_2_O, AR, 99.99%) were purchased from Aladdin (Shanghai, China). Hydrochloric acid (HCl, AR, 37%) was purchased from Sinopharm Chemical Reagent (SCR) (Shanghai, China). TC (USP) was purchased from SAITONG. Ferric chloride hexahydrate (FeCl_3_·6H_2_O, AR, 97%) was purchased from Alfa Aesar ((Haverhill, MA, USA)). All chemical reagents were used without further purification and the solutions were prepared with ultrapure water (18.2 MΩ cm) from a Millipore Simplicity water purification system (Shanghai, China).

### 2.2. Preparation of MnO_2_@fiber Tubular Micromotors

The obtained KFs were cut to lengths of 100–200 μm with scissors and treated with oxygen plasma to improve their surface hydrophilicity. A total of 1.12 g KFs were added to 60 mL of 1 mol L^−1^ KMnO_4_ solution at room temperature and pressure, followed by mild magnetic agitation. Agitation was used to ensure good dispersion of KFs in solution. After complete dispersion, the reaction was allowed to proceed for 72 h. Then, after centrifugation, the products were collected and cleaned three times each with deionized water and anhydrous ethanol, and then vacuum dried.

### 2.3. Preparation of MnFe_2_O_4_ Nanoparticles

MnFe_2_O_4_ NPs were synthesized by a hydrothermal method. A total of 2 g FeCl_3_·6H_2_O and 0.752 g MnCl_2_·4H_2_O were added to 70 mL glycol and dissolved by ultrasound. After that, 5 g sodium acetate and 3 g polyethylene glycol were added to the reaction system and then stirred for 1 h. After stirring, the mixture was transferred to a Teflon-lined reactor for 10 h at 200 °C. After the reaction, centrifugal collection was carried out, and the products were washed with deionized water and anhydrous ethanol three times respectively, and then dried at 60 °C for 8 h.

### 2.4. Preparation of MnFe_2_O_4_@MnO_2_@fiber Micromotors

The 10 mg MnO_2_@fiber tubular micromotors prepared as described above were added to 25 mL of 0.01 mol L^−1^ MnFe_2_O_4_ nanoparticle suspension. The resulting mixture was sealed and gently shaken to avoid agglomeration and to make the deposition as uniform as possible. After shaking for 24 h, centrifugal collection was performed and the products were cleaned with deionized water, followed by vacuum drying.

### 2.5. Motion Study of Micromotors

Experiments to investigate the motion of the micromotors were performed in solutions containing 0.5, 1.0, 1.5, and 2.0 wt% H_2_O_2_ with a fixed SDS concentration of 0.2 wt%. A mixture containing micromotors, H_2_O_2_, and SDS was dropped onto a slide. The speeds of the micromotors were analyzed by an OLYMPUS cellSens Dimension system. A commercial magnet was used to provide an external magnetic field for magnetic control of the micromotors. All optical images and video were taken by a microscope equipped with a camera. Speed tracking data were averaged from 10 independent experiments.

### 2.6. Preparation of Contaminated Soil

Soil samples used in the experiment were taken from the campus of China University of Mining and Technology. The soil was air-dried, ground, screened with a 2 mm sieve, and stored in a cool dry place for later use. Contaminated soil was prepared by adding a configured TC solution to the soil, and the final concentration of TC in the soil was 400 mg kg^−1^. The contaminated soil was aged for a week before being used in the experiment.

### 2.7. Extraction of TC from Soil

Briefly, a 2 g soil sample was placed in a centrifuge tube and 40 mL McIlvaine-Na_2_EDTA and acetonitrile (*v*:*v* = 1:2) were added as extractants. After vortexing for 1 min, ultrasonic extraction for 10 min, and then centrifugation for 10 min at 6000 r/min, the supernatant was collected. Finally, the sample was filtered through a 0.22 µm filter and the concentration of TC (λ = 355 nm) was measured using a UV-visible spectrophotometer.

### 2.8. Degradation of TC

The degradation of TC was carried out in a 100 mL beaker with 5 g contaminated soil and 50 mL deionized water for 30 min at room temperature. The soil and water mixture were constituted of TC (400 mg kg^−1^), micromotors (10 mg g^−1^), H_2_O_2_ (1.0 wt%), and PMS (5 × 10^−3^ M) to perform the degradation. Within the specified time interval, 1 mL solution was collected by a syringe, and supernatant was collected after extraction through a 0.22 µm filter. The resulting solution was immediately measured to determine the concentration of TC (λ = 355 nm) using a UV-vis spectrometer. The degradation efficiency of TC can be calculated by the following Equation (1):

(1) $$Degradation\;efficiency\;(\%)=\frac{C_{0}-C}{C_{0}}$$

The effects of soil-water ratio, micromotor dosages, H_2_O_2_ concentration, and pH value were investigated in the same reaction system at room temperature. The solution pH was adjusted by 0.01 M H_2_SO_4_ or NaOH. In addition, two sets of free radical quenching experiments were conducted to determine the radical species formed in the catalytic system using TBA and ethanol as radical scavengers. The experiments were carried out under the same experimental conditions.

### 2.9. Adsorption of Heavy Metal Ions

Magnetic micromotors (30 mg) were added to two 50 mL Pb^2+^ solutions (5000 ppb), one of which contained 1.0 wt% H_2_O_2_. A 0.1 M solution of HCl was used to adjust the pH of the solution to 4.0–5.4. At the specified time interval, 5 mL of solution was extracted using a syringe, and the supernatant was collected after centrifugation (12,000 rpm, 5 min). The concentration of Pb^2+^ in the solution was determined by inductively coupled plasma-mass spectrometry (ICP-MS). The adsorption capacity was calculated as follows:

(2) $$q_{e}=\frac{(C_{0}-C_{e})V}{m}$$

where *q_e_* is the adsorption capacity of lead ions (mg g^−1^), *C*_0_ and *C_e_* are the initial and equilibrium concentrations of lead ions (mg L^−1^), respectively, *V* is the working volume (L), and *m* is the weight of the adsorbent (g).

## 3. Results and Discussion

### 3.1. Fabrication and Characterizations of Micromotors

**Figure 1:**
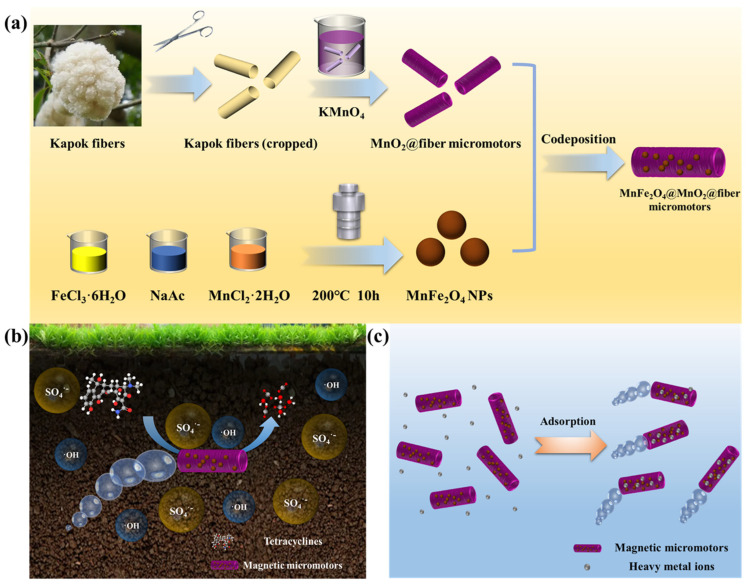
(**a**) Preparation of magnetic micromotors. (**b**) Schematic diagram of the magnetic micromotors as a heterogeneous catalyst for degradation of antibiotics. (**c**) Schematic diagram of the magnetic micromotors as an adsorbent for adsorption of heavy metal ions.

As a plant fiber, Kapok fibers with a hollow tubular structure exist widely in nature and are easy to obtain at low cost and in large quantities, providing ideal biological templates for the fabrication of tubular micromotors [23]. In addition, manganese dioxide was used in the construction of the micromotors due to its advantages of low cost, environmental friendliness, and excellent catalytic properties. The fabrication procedures are schematically depicted in Figure 1a. Prior to the manufacturing process, raw Kapok fibers were cut with scissors to suitable lengths and treated with oxygen plasma to achieve the desired hydrophilicity, which is favorable for their subsequent dispersion in KMnO_4_ solution to deposit MnO_2_ on the surface. Since KMnO_4_ can react with cellulose and hemicellulose, the MnO_2_ nanoparticles will be grown on the walls of the fibers. Then, the MnFe_2_O_4_ nanoparticles prepared by a hydrothermal method were anchored on the surface of MnO_2_@fiber micromotors via co-deposition to yield MnFe_2_O_4_@ MnO_2_@fiber micromotors. The widely-used oxidant H_2_O_2_ serves as both the fuel to propel the micromotors and the precursor of active species. As demonstrated in Figure 1b, as well as displaying autonomous motion, the micromotors can generate SO_4_^•−^ and •OH via activation of PMS and H_2_O_2_ to degrade antibiotics. Additionally, since the prepared micromotors had a relatively high negative zeta potential, they can perform on-the-fly adsorption of heavy metals in the environment (Figure 1c) [24].

**Figure 2:**
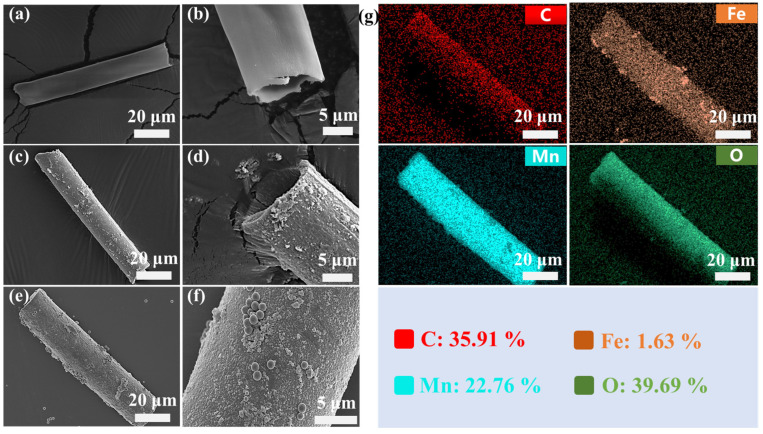
SEM images of (**a**,**b**) kapok fiber, (**c**,**d**) KF@MnO_2_ micromotors, (**e**,**f**) magnetic micromotors, and (**g**) corresponding EDX elemental mapping.

The morphology of the Kapok fiber, MnO_2_@fiber micromotors, and MnFe_2_O_4_@MnO_2_@fiber micromotors was characterized by scanning electron microscopy (SEM) (Figure 2a–f). The components of the MnFe_2_O_4_@MnO_2_@fiber micromotors were also analyzed by Energy-dispersive X-ray spectroscopy (EDX) mapping. It can be seen that the fibers present a hollow tubular structure with an average length of 170 μm and an opening diameter of 17 μm (Figure 2a,b). The MnO_2_ nanoparticles formed via immersion in KMnO_4_ solution can be observed on both the inner and outer surfaces of the microtubes, as shown in Figure 2c,d, while the original tubular structure of the fibers was maintained. Figure 2e–g show the SEM image and corresponding EDX elemental mapping of the MnFe_2_O_4_@MnO_2_@fiber micromotors. As displayed in the zoomed-in image of the micromotor surface in Figure 2f, the spherical MnFe_2_O_4_ NPs were successfully integrated on the surface of the micromotor. The EDX elemental mapping in Figure 2g confirmed that the micromotor contained C, Fe, Mn, and O elements, demonstrating that the hollow fibers were uniformly modified with MnO_2_ NPs and MnFe_2_O_4_ NPs. Compared to the traditional template-assisted electrodeposition method and rolling-up method to prepare tubular micromotors, the simple and environmentally friendly preparation process described here is more cost-effective [25]. Meanwhile, the natural materials and nontoxic materials used in the fabrication avoid secondary pollution to the environment during the execution of tasks.

**Figure 3:**
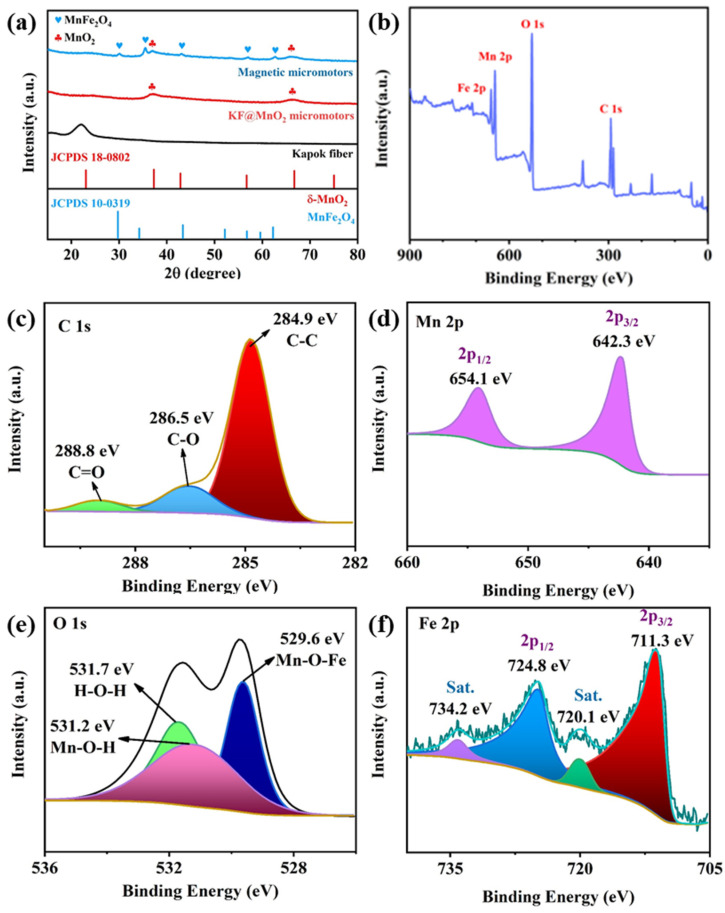
(**a**) XRD patterns of Kapok fibers, MnO_2_@fiber micromotors, and MnFe_2_O_4_@MnO_2_@fiber micromotors. (**b**) XPS spectrum of MnFe_2_O_4_@MnO_2_@fiber micromotors. XPS spectra of (**c**) C 1s, (**d**) Mn 2p, (**e**) O 1s, and (**f**) Fe 2p.

In order to further investigate the composition of the micromotors, X-ray Diffraction (XRD) patterns of the Kapok fibers, MnO_2_@fiber micromotors, and MnFe_2_O_4_@MnO_2_@fiber micromotors were acquired (Figure 3a). The wide diffraction peak at 22° indicated a typical cellulosic I structure of the Kapok fibers. The two weak diffraction peaks located at 36.9° and 66.2°, which can be indexed to the (0 0 6) and (1 1 9) planes, proved that the fibers were modified with the birnessite-type MnO_2_ (JCPDS 18-0802, δ-MnO_2_) via reduction of KMnO_4_ [26]. The diffraction peaks located at 30.0°, 35.5°, 43.0°, 56.9°, and 62.7° corresponded to the (2 2 0), (3 1 1), (4 0 0), (5 1 1), and (4 4 0) planes of the MnFe_2_O_4_ cubic crystal structure, respectively (JCPDS 10-0319) [27]. The XRD results showed that MnO_2_ NPs and MnFe_2_O_4_ NPs were successfully integrated on the surface of the fibers. In addition, the elemental valences of the magnetic micromotors have been studied by X-ray photoelectron spectroscopy (XPS). As shown in Figure 3b, the XPS spectrum clearly certifies the presence of four elements (C, Fe, Mn, and O). Figure 3c–f show the XPS spectra of C 1s, Mn 2p, O 1s, and Fe 2p, respectively. In Figure 3c, three characteristic peaks at 284.9 eV, 286.5 eV, and 288.8 eV can be obtained by deconvolution of the C 1s peak, corresponding to the C-C sp_2_ peak, C-O bond, and C=O bond, respectively [28]. As shown in Figure 3d, the two peaks at 642.3 and 654.1 eV can be assigned to Mn 2p_3/2_ and Mn 2p_1/2_, respectively, and the splitting energy was 11.8 eV, indicating the predominant presence of Mn^4+^ [29]. Figure 3e shows the presence of three types of oxygen-containing chemical bonds, namely Mn-O-Fe at 529.6 eV, M-O-H at 531.2 eV, and H-O-H (water molecules) at 531.7 eV [30]. The Fe 2p spectrum consists of two sublevels, Fe 2p_3/2_ at 711.3 eV and Fe 2p_1/2_ at 724.8 eV, as is shown in Figure 3f. At the same time, a Fe 2p_3/2_ satellite peak can be observed at 720.1 eV, which confirms the presence of Fe^3+^ in the magnetic micromotors and provides evidence for the successful growth of MnFe_2_O_4_ NPs onto the micromotors [31]. The XPS results clearly demonstrate the successful synthesis of MnFe_2_O_4_@MnO_2_@fiber micromotors.

### 3.2. The Motion Behaviors of Magnetic Micromotors

**Figure 4:**
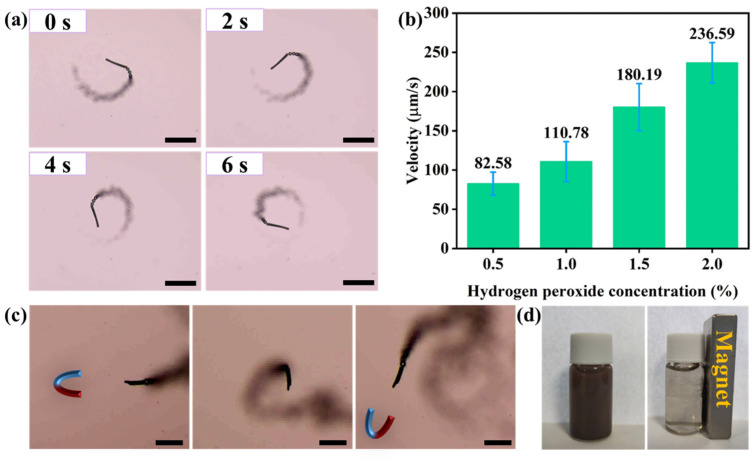
(**a**) Time-lapse image of the motion of a micromotor in 1 wt% H_2_O_2_. Scale bars are 100 µm. (**b**) Velocities of micromotors at different H_2_O_2_ concentrations. A total of 10 independent experiments were conducted to obtain the average velocity. The error bars represent the standard deviation. (**c**) Magnetic guidance of a micromotor. Scale bars are 100 µm. (**d**) Magnetic separation of the MnFe_2_O_4_@MnO_2_@fiber micromotors.

The propulsion of the prepared micromotors originates from the decomposition of H_2_O_2_ into oxygen and water catalyzed by MnO_2_. The oxygen bubbles generated asymmetrically push the micromotor in the opposite direction. Moreover, H_2_O_2_ is not only the fuel of micromotor propulsion but also the indispensable reagent for subsequent Fenton/Fenton-like reactions. Figure 4a shows the motion of a micromotor in an aqueous solution containing 1.0 wt% H_2_O_2_ (SI Video S1). The MnO_2_ NPs inside the microtube wall of the micromotor continuously decompose H_2_O_2_ to produce oxygen bubbles, resulting in a tail of bubbles behind the micromotor. Although the MnO_2_ NPs grow on both the inner and outer walls of the micromotor, oxygen bubbles tend to be generated on the inner walls and ejected at only one end to push the micromotor forward, which is related to the difference in the Laplace pressure at the two sides of the bubble generated inside the microtube [32,33]. As observed in Figure 4b, with an increase in H_2_O_2_ concentration, the motion speed of the micromotor also increases. In the solution of 0.5 wt%, 1 wt%, 1.5 wt%, and 2 wt% H_2_O_2_, the average velocity of micromotors motion reached 82.58 μm/s, 110.78 μm/s, 180.19 μm/s, and 236.59 μm/s, respectively. In the presence of Tween 20, the prepared micromotors can also display efficient movement as shown in Figure S1.

In addition, due to the magnetic responsiveness of the MnFe_2_O_4_ NPs, the micromotors loaded with MnFe_2_O_4_ NPs can achieve directional motion under the guidance of an external magnetic field. Upon applying an external magnetic field, the micromotor moves in a straight line along the direction of the magnetic field. Once the external magnetic field is removed, the motion of the micromotor changes to random motion or circular motion. When the magnetic field is applied again, the micromotor moves according to the direction of the magnetic field again (Figure 4c, SI Video S2). As shown in Figure 4d, the use of magnets allows the rapid separation and collection of the micromotors, which allows convenient recycling of the micromotors after use and avoids potential secondary pollution to the environment. The magnetic guidance also provides possibilities for micromotors to perform tasks in tiny and narrow spaces that conventional tools cannot reach.

### 3.3. Degradation Performance of Micromotors

**Figure 5:**
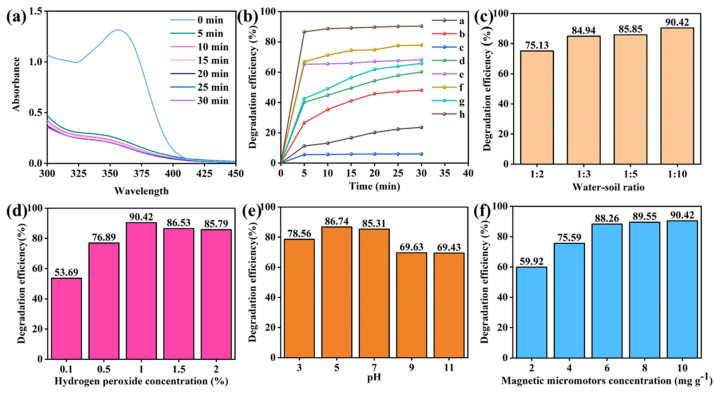
(**a**) UV-vis absorption spectra of tetracycline (TC) in the presence of magnetic micromotors with 1.0 wt% H_2_O_2_ and 5 × 10^−3^ M PMS at different time intervals. (**b**) Degradation efficiency of TC of different systems: (a) H_2_O_2_, (b) PMS, (c) magnetic micromotors, (d) KF@MnO_2_ micromotors + H_2_O_2_, (e) KF@MnO_2_ micromotors + PMS, (f) KF@MnO_2_ micromotors + H_2_O_2_ + PMS, (g) MnFe_2_O_4_ NPs + H_2_O_2_ + PMS, (h) magnetic micromotors + H_2_O_2_ + PMS. (**c**) The effect of water–soil ratio on TC degradation efficiency. (**d**) The effect of H_2_O_2_ concentration on TC degradation efficiency. (**e**) The effect of pH on TC degradation efficiency. (**f**) The effect of magnetic micromotor concentration on TC degradation efficiency.

Antibiotics have been detected in soil samples from many countries around the world. Therefore, it is of great practical significance to investigate new and efficient antibiotic degradation technology. Here, TC was employed as the target pollutant to explore the catalytic performance of the prepared micromotors as heterogeneous catalysts to degrade pollutants. As shown in Figure 5a, after the magnetic micromotors were added to the reaction system for 5 min, the absorption peak of TC dropped sharply, indicating that the micromotors possess catalytic activity toward TC degradation. Afterward, the content of TC in the system was further reduced and reached a degradation efficiency of 90.4% at 30 min. The degradation experiments were conducted in different systems to explore the function of various components. As Figure 5b shows, when only 1 wt% H_2_O_2_ was present in the reaction system (curve a), only 23.6% of TC was degraded within 30 min. When only PMS was present in the reaction system (curve b), the degradation efficiency was slightly better, reaching 48.1%. The adsorption effect of magnetic micromotors in TC removal was insignificant (curve c). Next, the catalytic performances of MnO_2_@fiber micromotors in single H_2_O_2_ or PMS systems were investigated, respectively. With the addition of micromotors into the H_2_O_2_ system, the TC degradation efficiency increased sharply to 60.2% (curve d), which proved the generation of more active radicals from H_2_O_2_-catalyzed micromotors. Furthermore, the mass transfer enhancement of the reaction system caused by the propulsion of the micromotors as well as a large number of bubbles is also conducive to the degradation of pollutants. Moreover, as a comparison of curves b and e in Figure 5b shows, when micromotors were added to the PMS system, the TC degradation efficiency increased by 20% on the basis of a single PMS system, which also proved the activation ability of the micromotors to PMS. Subsequently, when the MnO_2_@fiber micromotors were added to the combined system of H_2_O_2_ and PMS, the TC degradation efficiency was further increased (curve f). In addition, the catalytic activity of MnFe_2_O_4_ NPs and magnetic micromotors were also tested in the combined system of H_2_O_2_ and PMS. The magnetic micromotors showed the best catalytic performance, with a TC removal efficiency exceeding 90%. The higher catalytic activity of the magnetic micromotors compared to the MnO_2_@fiber micromotors can be attributed to the synergistic effect of MnFe_2_O_4_ and MnO_2_. To verify the applicability of the prepared micromotors for the degradation of other organic pollutants, degradation experiments of Rhodamine B and salicylhydroxamic acid by the system were conducted (Figures S2 and S3) and degradation efficiencies of more than 95% could be achieved for both contaminants within 30 min.

Compared with micromotors containing only MnO_2_ catalyst, the MnFe_2_O_4_ NPs component in the magnetic micromotors can also trigger Fenton-like reactions through the continuous conversion of Fe^3+^ and Fe^2+^ to generate more radicals to achieve efficient degradation of antibiotics. The process is shown in the following Equations (3)–(12) [34,35]:

Fe^3+^ + H_2_O_2_ → Fe^2+^ + HOO• + H^+^ (3)

Fe^2+^ + H_2_O_2_ → Fe^3+^ + •OH + OH^−^ (4)

Fe^3+^ + HSO_5_^−^ → Fe^2+^ + SO_5_^•−^ + H^+^ (5)

Fe^2+^ + HSO_5_^−^ → Fe^3+^ + SO_4_^•−^ + OH^−^ (6)

Mn^4+^ + HSO_5_^−^ → Mn^3+^ + SO_5_^•−^ + H^+^ (7)

Mn^3+^ + HSO_5_^−^ → Mn^2+^ + SO_5_^•−^ + H^+^ (8)

Mn^3+^ + HSO_5_^−^ → Mn^4+^ + SO_4_^•−^ + OH^−^ (9)

Mn^2+^ + HSO_5_^−^ → Mn^3+^ + SO_4_^•−^ + OH^−^ (10)

2SO_5_^•−^ → 2SO_4_^•−^ + O_2_ (11)

SO_4_^•−^ + H_2_O → •OH + SO_4_^2−^ + H^+^ (12)

Next, the effects of water–soil ratio, hydrogen peroxide concentration, pH, and magnetic micromotor concentration on TC degradation efficiency were further explored. As shown in Figure 5c, the degradation efficiency increased from 75.13% to 90.42% with an increase in water content in the reaction system, indicating that an increase in water content is conducive to the degradation. When the concentration of hydrogen peroxide increased from 0.1 wt% to 2 wt% (Figure 5d), the TC degradation efficiency first increased and then, when the concentration exceeded 1 wt%, slightly decreased. It can be seen from Figure 5e that from pH 3 to 7, the degradation efficiency slightly increased. This effect may be attributed to the fact that H_2_O_2_ will solvate protons to form H_3_O_2_^+^ under relatively strong acidic conditions, which will affect the Fenton-like reaction to produce active radicals. In addition, HSO_5_^−^ in PMS will also form H_2_SO_5_ under acidic conditions, which is not conducive to the production of SO_4_^•−^. When the pH was above 7, the TC degradation efficiency decreased by more than 20%, as in alkaline environments, the Fenton reaction was greatly inhibited. At the same time, the reaction of Fe^2+^ with hydroxide ion (OH^−^) under alkaline conditions produced colloidal precipitation, which also influenced the formation of active radicals. In addition, the main existing form of PMS, HSO_5_^−^, reacted with OH^−^ to form SO_5_^2−^, hindering the production of SO_4_^•−^. As expected, the degradation efficiency gradually increased as the concentration of the magnetic micromotors increased. As shown in Figure 5f, when the concentration of the magnetic micromotors increased from 2 to 6 mg g^−1^, the degradation efficiency was elevated from 59.92% to 88.26%. Upon reaching 6 mg g^−1^, the increase in degradation efficiency slowed.

**Figure 6:**
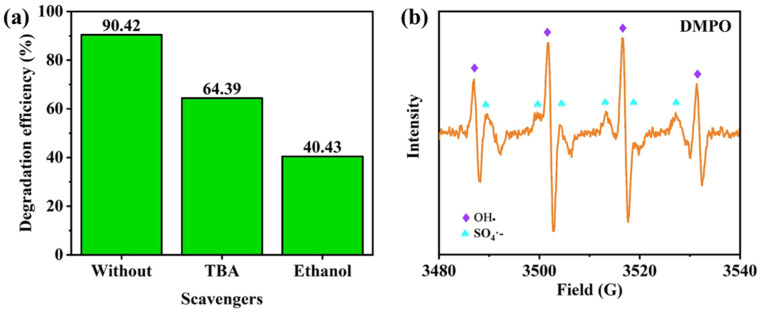
(**a**) Effect of radical scavengers on TC degradation. (**b**) EPR spectra using DMPO spin capturing.

To evaluate the active species in the magnetic micromotors/H_2_O_2_/PMS system, free radical trapping experiments were conducted, as shown in Figure 6a. Ethanol was used as the trapping agent of SO_4_^•−^, •OH and TBA was used to trap •OH but not SO_4_^•−^. When TBA and ethanol were added into the reaction system, the TC degradation efficiency decreased by 26.03% and 49.99%, respectively, within 30 min. The results show that both •OH and SO_4_^•−^ participate in TC degradation and have significant contributions. To further verify the reactive species that play a role in the reaction system, electron paramagnetic resonance (EPR) measurements were performed (Figure 6b). Four-line spectral peaks (1-2-2-1) and six-line spectral peaks (1-1-1-1-1-1) appear in the EPR spectra, indicating that the magnetic micromotors can activate H_2_O_2_ and PMS to produce •OH and SO_4_^•−^ [36,37]. Based on the above experimental results, it can be confirmed that both •OH and SO_4_^•−^ exist in the reaction system and contribute to TC degradation.

### 3.4. Adsorption Capacity of Magnetic Micromotors

**Figure 7:**
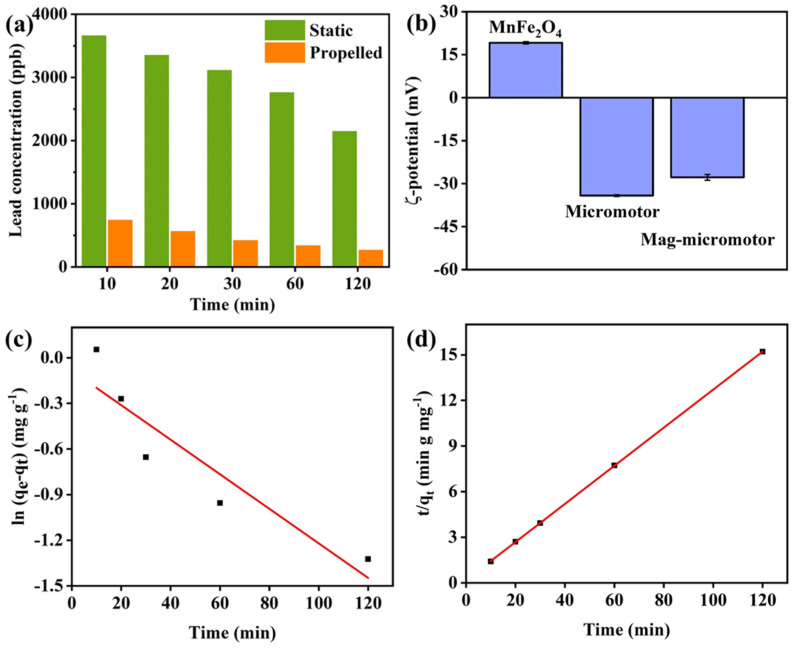
(**a**) Lead concentration in the presence of the static group and self-propelled group of micromotors. (**b**) ζ-potential values of MnFe_2_O_4_ NPs, KF@MnO_2_ micromotors, and magnetic micromotors. (**c**) Pseudo-first-order and (**d**) pseudo-second-order kinetic models for fitting results of self-propelled group.

Considering the large specific surface area, abundant active sites, and surface charge properties of the magnetic micromotors, the ability of the magnetic micromotors to adsorb heavy metal ions was experimentally investigated, as shown in Figure 7a. During the preparation process, a large number of polar groups such as hydroxyl, carboxyl, and amino groups were introduced on the surface of the fibers after oxygen plasma treatment [38,39]. These functional groups enhance the adsorption of heavy metal ions on micromotors through electrostatic attraction and complex formation. Control experiments using static micromotors were also performed for comparison. During the same time interval, compared with static micromotors, the self-propelled micromotors exhibited appreciably improved adsorption of lead, which proves the positive effect of self-propulsion on heavy metal removal. When the adsorption time reached 120 min, the adsorption efficiency of the self-propelled group was more than 94%, while that of the static group was only 57%. Previous studies established that the surface charge of the adsorbent influences ion adsorption [40]. Through a zeta potential test (Figure 7b), it was determined that the surface of the magnetic micromotors has a strong negative charge, which is favorable for the adsorption of heavy metal cations. Due to self-propulsion as well as the strong electrostatic repulsion between highly negatively charged surfaces, the aggregation of magnetic micromotors in aqueous solution can be inhibited, thus providing more adsorption sites for the removal of heavy metal ions. The enhanced mass transfer induced by the self-propulsion and O_2_ bubble generation also increases the contact probability of heavy metal ions and the micromotors, thus improving the adsorption efficiency of the self-propelled micromotors. The distribution of lead elements in the tubular micromotors, as shown in the EDX results in Figure S4, confirms their adsorption by the magnetic micromotors. In addition, the micromotors maintained their tubular structure after the adsorption and degradation experiments, which proves the good mechanical stability of the prepared micromotors (Figure S5).

In order to further explore the adsorption mechanism, a study of the adsorption kinetics was conducted. Based on the pseudo-first-order (Equation (13)) and pseudo-second-order kinetic models (Equation (14)), the adsorption kinetics curves were plotted (Figure 7c,d).

(13) $$\ln\left(q_{e}-q_{t}\right)=lnq_{e}-k_{1}t$$

(14) $$\frac{t}{q_{t}}-\frac{1}{k_{2}q_{e}^{2}}=\frac{t}{q_{e}}$$

where *q_e_* is the adsorption capacity of lead ions (mg g^−1^), *q_t_* is the adsorption of lead ions at time t, and *k*_1_ and *k*_2_ are the rate constant of the pseudo-first-order and pseudo-second-order dynamics models, respectively. The correlation coefficient (R^2^) was used to determine the fitting of the kinetic models. The results showed that the pseudo-second-order kinetic model (R^2^ = 0.99998) had better fitting results than the pseudo-first-order kinetic model (R^2^ = 0.81036). This indicates that the chemical interaction was the main rate-controlling step in the adsorption of lead by magnetic micromotors.

## 4. Conclusions

In summary, we have developed bubble-propelled tubular micromotors, which can be used as active heterogeneous catalysts for the efficient degradation of antibiotics and as adsorbents for the removal of heavy metals. The magnetic micromotors exhibit rapid self-propulsion in H_2_O_2_ solution, and the mass transfer enhancement caused by autonomous movement and microbubble generation overcomes the shortcomings of traditional heterogeneous catalysts, which achieve low degradation efficiency due to low diffusion rates. By coupling MnO_2_ with MnFe_2_O_4_, the micromotors could activate H_2_O_2_ and PMS to produce •OH and SO_4_^•−^, leading to a TC degradation efficiency of more than 90% in soil within 30 min. In addition, their large specific surface area, active groups on the surface, and negative surface charge enable the micromotors to efficiently adsorb lead ions, resulting in the removal of more than 94% of lead ions in water within 120 min. After the completion of the desired tasks, the micromotors can be recovered by the application of an external magnetic field, which avoids secondary pollution caused by the catalyst and adsorbent and provides the possibility of multiple recycling. Overall, the bioinspired micromotors based on natural fibers offer a new platform for the degradation of organic pollutants as well as the removal of heavy metals.

## Data Availability

The data that support the findings of this study are available on request from the corresponding author. The data are not publicly available due to privacy or ethical restrictions.

## Supplementary Materials

The following supporting information can be downloaded at: https://www.mdpi.com/article/10.3390/nano13192710/s1, Figure S1: Time-lapse image of the motion of a magnetic micromotor in 1 wt% H_2_O_2_ and 0.2 wt% Tween 20. Figure S2: The degradation efficiency of Rhodamine B. Figure S3: The degradation efficiency of salicylhydroxamic acid. Figure S4: The SEM image of magnetic micromotors after adsorption of Pb^2+^ and corresponding EDX elemental mapping. Figure S5: The SEM image of magnetic micromotors after the adsorption and degradation experiments. Video S1: The random motion of a magnetic micromotor in 1 wt% H_2_O_2_ and 0.2 wt% SDS. Video S2: The magnetically guided motion of a magnetic micromotor in 1 wt% H_2_O_2_ and 0.2 wt% SDS.
