# Supplementary material for: Multifunctional Biotemplated Micromotors for In Situ Decontamination of Antibiotics and Heavy Metals in Soil and Groundwater
Source: Nanomaterials (Basel). 2023 Oct 6;13(19):2710. doi: 10.3390/nano13192710 (PMC10574631; doi:10.3390/nano13192710)
Supplement: Supplementary file 1 [file nanomaterials-13-02710-s001.zip › Support Information-0920.pdf]

Supporting Information

Multifunctional Biotemplated Micromotors for in-situ Decontamination of Antibiotics and Heavy Metals in Soil and Groundwater

Haohao Cui, Ke Wang , Enhui Ma and Hong Wang *

Figure S1. Time-lapse image of the motion of a magnetic micromotor in 1 wt% H_2O_2 and 0.2 wt% Tween 20.

Figure S2. The degradation efficiency of Rhodamine B at magnetic micromotors + H_2O_2 + PMS system.

Figure S3. The degradation efficiency of salicylhydroxamic acid at magnetic micromotors + H_2O_2 + PMS system.

Figure S4. The SEM images of magnetic micromotors after adsorption of Pb^{2+} and corresponding EDX elemental mapping.

Figure S5. The SEM image of magnetic micromotors after degradation of TC and adsorption of Pb^{2+}.

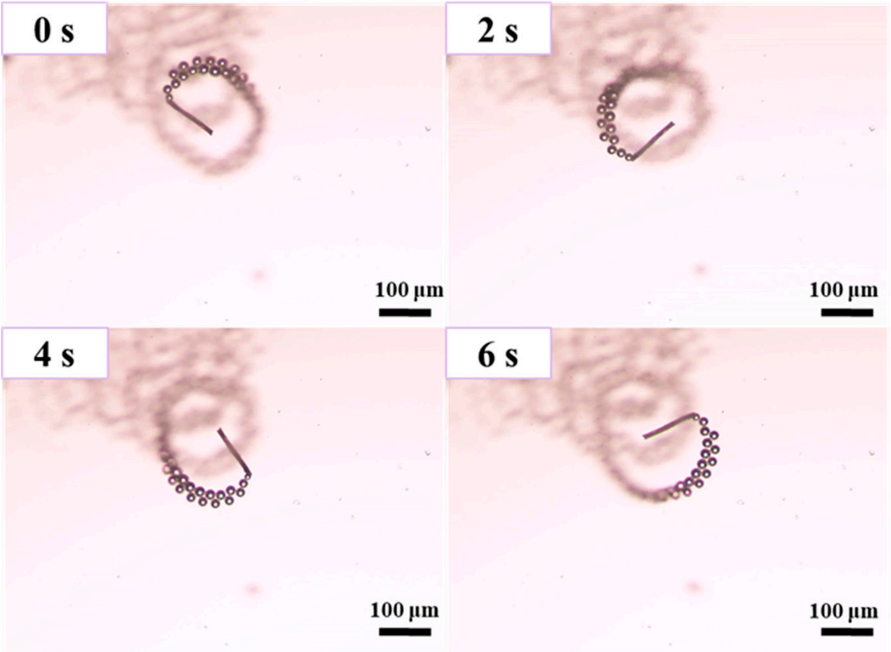

Figure S1. Time-lapse image of the motion of a magnetic micromotor in 1 wt% H_2O_2 and 0.2 wt% Tween 20.

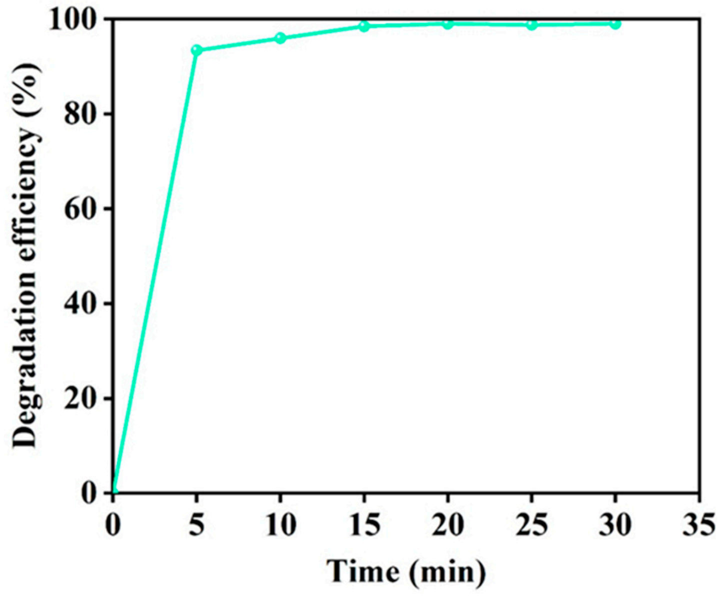

Figure S2. The degradation efficiency of Rhodamine B at magnetic micromotors + H_2O_2 + PMS system.

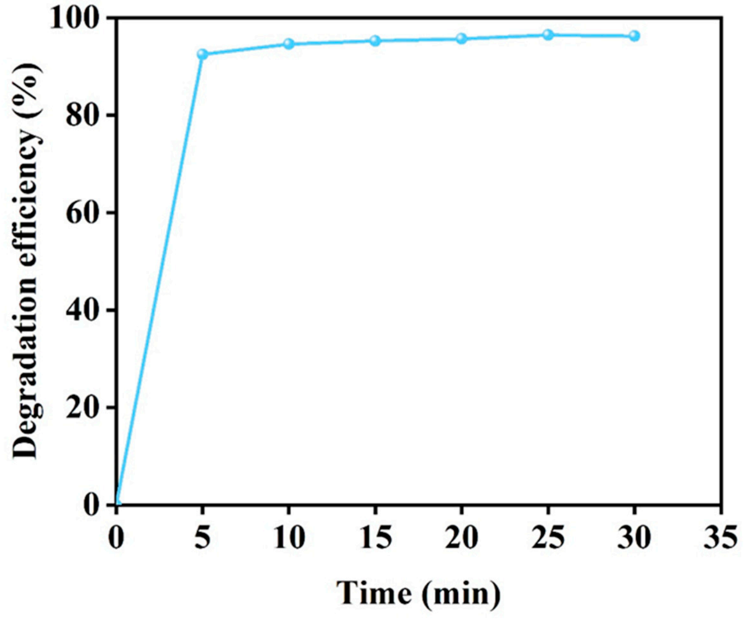

Figure S3. The degradation efficiency of salicylhydroxamic acid at magnetic micromotors + H_2O_2 + PMS system.

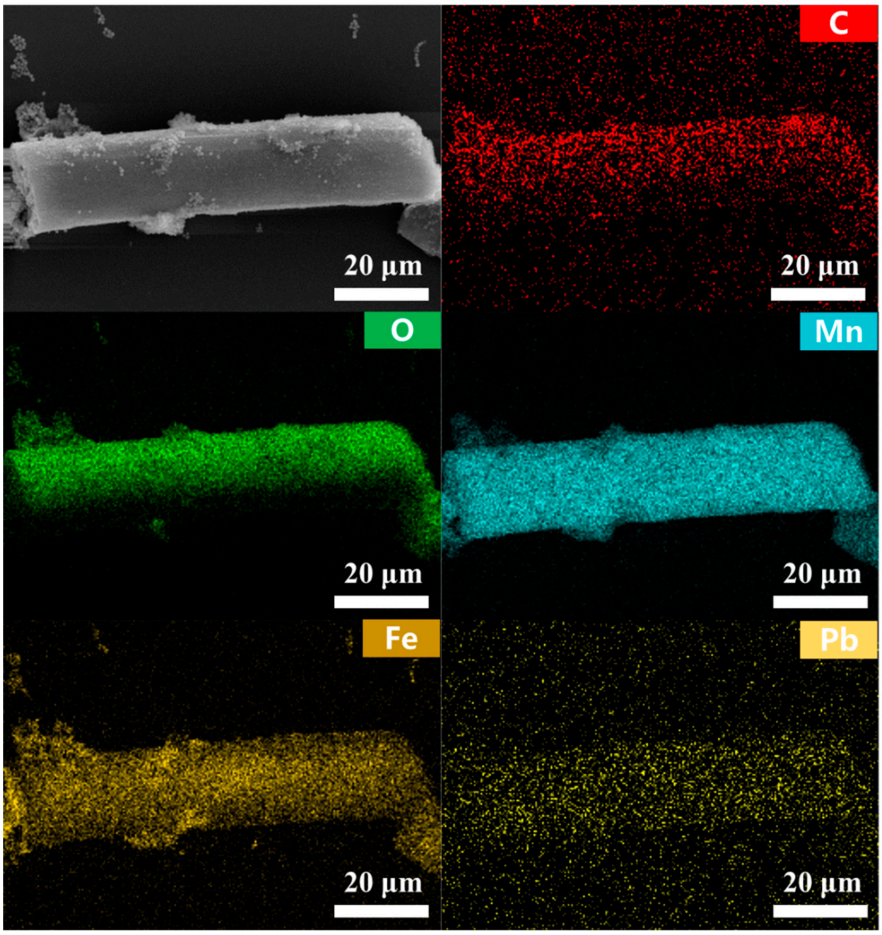

Figure S4. The SEM image of magnetic micromotors after adsorption of Pb^{2+} and corresponding EDX elemental mapping.

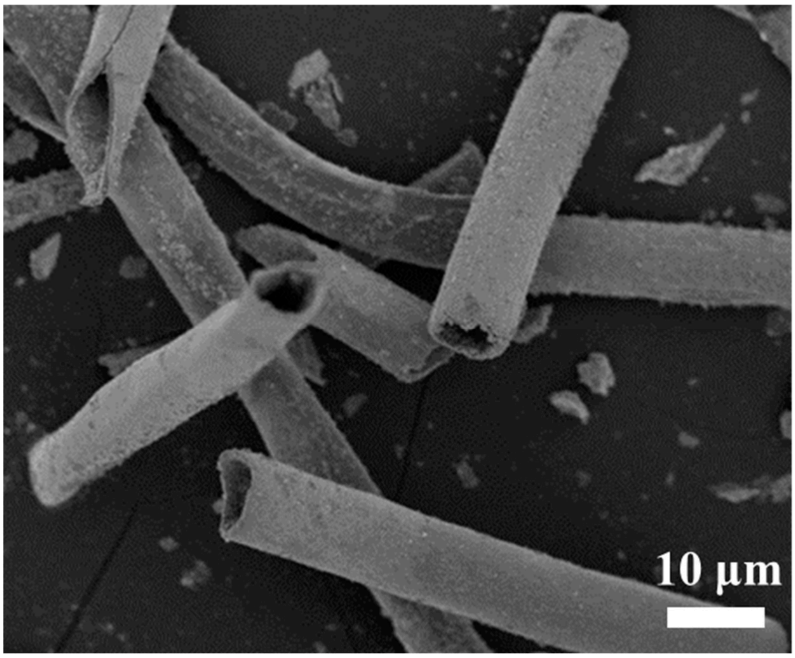

Figure S5. The SEM image of magnetic micromotors after degradation of TC and adsorption of Pb^{2+}
